# Synergistic Cytoprotective Effects of Rutin and Ascorbic Acid on the Proteomic Profile of 3D-Cultured Keratinocytes Exposed to UVA or UVB Radiation

**DOI:** 10.3390/nu11112672

**Published:** 2019-11-05

**Authors:** Agnieszka Gęgotek, Iwona Jarocka-Karpowicz, Elżbieta Skrzydlewska

**Affiliations:** Department of Analytical Chemistry, Medical University of Bialystok, 15-089 Bialystok, Poland; iwona.jarocka-karpowicz@umb.edu.pl (I.J.-K.); elzbieta.skrzydlewska@umb.edu.pl (E.S.)

**Keywords:** keratinocytes, rutin, ascorbic acid, UV radiation, proteomics, 3D cell culture

## Abstract

The combination of ascorbic acid and rutin, often used in oral preparations, due to antioxidant and anti-inflammatory properties, can be used to protect skin cells against the effects of UV radiation from sunlight. Therefore, the aim of this study was to investigate the synergistic effect of rutin and ascorbic acid on the proteomic profile of UVA and UVB irradiated keratinocytes cultured in a three-dimensional (3D) system. Results showed that the combination of rutin and ascorbic acid protects skin cells against UV-induced changes. In particular, alterations were observed in the expression of proteins involved in the antioxidant response, DNA repairing, inflammation, apoptosis, and protein biosynthesis. The combination of rutin and ascorbic acid also showed a stronger cytoprotective effect than when using either compound alone. Significant differences were visible between rutin and ascorbic acid single treatments in the case of protein carboxymethylation/carboxyethylation. Ascorbic acid prevented UV or rutin-induced protein modifications. Therefore, the synergistic effect of rutin and ascorbic acid creates a potentially effective protective system against skin damages caused by UVA and UVB radiation.

## 1. Introduction

Maintaining the appropriate skin proteomic profile is a critical parameter for proper cell function and maintenance of healthy skin. For this reason, skin cells are characterized by the well-developed cytoprotective system, comprised of antioxidant, anti-inflammatory, and anti-apoptotic proteins. This protection system also involves active cytoprotective transcription factors which are responsible for protein biosynthesis [1]. However, despite these cytoprotective mechanisms, frequent exposure of skin cells to ultraviolet radiation (UV), mainly UVB (electromagnetic radiation with wavelength from 280 nm to 315 nm) and UVA (from 315 nm to 380 nm) contained in sunlight, stimulates pro-oxidant enzyme activity and impairs the action of antioxidants, resulting in oxidative stress [2,3]. As a consequence, UV enhances ROS-dependent modifications in skin cells covering all cellular components including nucleic acids, proteins, and lipids. This is particularly seen in lipid modification which causes an increase in the level of lipid peroxidation products, including 4-hydroxynonenal (4-HNE), which functions as an important signaling molecule [4]. In addition, the resulting highly reactive electrophilic aldehydes can interact with proteins and significantly modify their structure [5]. Also, UV-induced DNA damage activate proteins responsible for nucleic acids repairing [6]. On the other hand, in the case of keratinocytes, UVB radiation also enhances protein modifications such as advanced glycation end products (AGEs), including lysine carboxymethylation (CML) [7]. Generation of CML leads to dysfunction of the proteins biological functions, their translocation into the cell membrane, and activation of G-protein [8]. As a result, the expression of G-protein coupled receptors is often increased.

As a result, changes in the proteomic profile, including modifications of protein activity, are observed [9,10]. Previous studies have shown that skin cells exposure to UVA and UVB radiation leads to the activation of many factors involved in mitogen-activated protein (MAP)-dependent signaling kinases, including ERK1/2 and transcription factors dependent on redox potential, e.g., Nrf2 [11,12]. As a consequence, the described action of UV radiation leads to metabolic disorders in the skin which can significantly deteriorate the condition of the skin [13].

**Figure 1:**
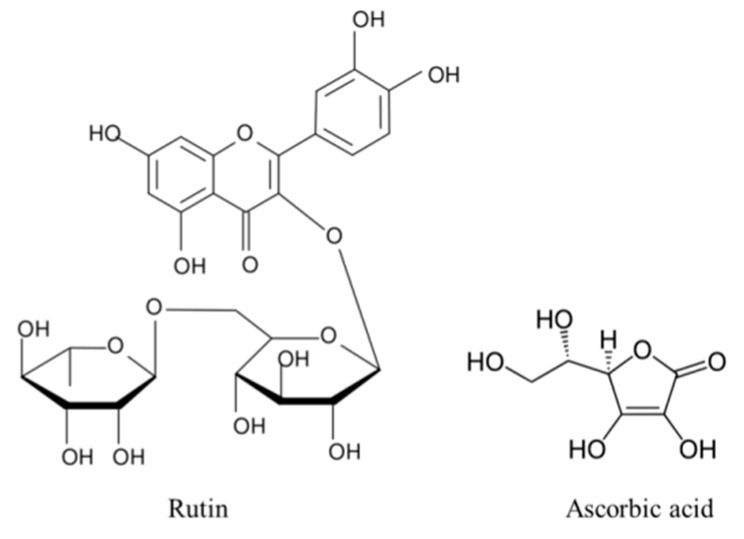
The comparison of chemical structure of rutin and ascorbic acid.

Due to the hazards of UV exposure, there is a constant need for natural, effective, daily use skin protection compounds. Examples of such compounds are rutin and ascorbic acid, not only due to their potent antioxidant properties resulting from their chemical structure (Figure 1), but also due to their synergistic cytoprotective activity [14]. It has been shown that rutin affects cellular metabolism not only via its antioxidant activity, but also by affecting the biological activity of proteins in varying signaling pathways. Rutin activates cytoprotective transcription factor Nrf2, and also inhibits the activity of cyclooxygenases and lipoxygenases, thereby reducing pro-inflammatory processes [15,16]. Additionally, rutin exerts cytoprotective effects on cells exposed to different types of radiation by substantially increasing their viability [17]. The use of rutin in in vitro cultures may be limited by its toxicity in a high concentration, however, different literature data provide different results regarding the range of its toxicity. In addition, this value depends on the type of cells as well as the length of treatment; long-term rutin treatment of cancer cells shows that the toxic concentration of rutin is already in the range 125–250 µM [18,19]. However, in the case of HaCaT keratinocytes, rutin toxicity is visible only above a concentration of 1 mM [20]. However, the action of rutin is limited by cell membrane permeability, which is significantly increased by UV radiation as a result of activation of membrane transporter, bilitranslocase [10]. Ascorbic acid also enhances rutin membrane permeability [21]. On the other hand, ascorbic acid applied to the skin exhibits a number of cytoprotective activities, such as supporting the antioxidant effect of vitamin E [22] and normalizing respiratory chain action by stabilizing mitochondrial membrane polarization in UV-irradiated human skin fibroblasts [23], as well as providing significant protection against inflammation and sunburn [24]. Treatment with ascorbic acid induces well-organized multilayers in 3D keratinocyte cultures, however, exceeding a concentration of 1 mM promotes too fast and uncontrolled cell differentiation [25,26].

To date, the interaction of orally-administered rutin with ascorbic acid has been demonstrated in terms of anti-inflammatory and vascular sealing actions [27]. Moreover, recent work has shown that combining rutin with ascorbic acid results in a synergistic, cytoprotective effect from UV radiation in vitro [14]. The aim of this study was to examine the combined effect of rutin and ascorbic acid on the proteomic profile in UVA and UVB irradiated keratinocytes grown in a three-dimensional (3D) epidermal-like system.

## 2. Materials and Methods

### 2.1. Cell Culture and Treatment

Human cell keratinocytes line CDD 1102 KERTr were obtained from American Type Culture Collection (ATCC, Manassas, VA, USA) and cultured in a humidified atmosphere of 5% CO_2_ at 37 °C. According to the cell culture protocol provided by ATCC, the growth medium for keratinocytes was keratinocyte-SFM medium supplemented with 1% bovine pituitary extract (BPE), human recombinant epidermal growth factor (hEGF), 50 μg/mL streptomycin, and 50 U/mL penicillin. Sterile and cell culture reagents were obtained from Gibco (Grand Island, NY, USA). 3D culture was carried out in AlgiMatrix plates (Life Technologies, Carlsbad, CA, USA).

Keratinocytes, following four days of 3D gel culturing, were irradiated with the following UV doses: UVA (365 nm)—30 J/cm^2^ and UVB (312 nm)—60 mJ/cm^2^ (Bio-Link Crosslinker BLX 312/365; Vilber Lourmat, Germany). To observe the effect of ascorbic acid and rutin on UV radiated cells following exposure to UV radiation, cells were incubated for 24 h under standard conditions in medium containing 100 µM ascorbic acid (19.81 mg/L) or/and 25 µM rutin (15.25 mg/L) in 0.1% DMSO. The concentration of ascorbic acid was selected according to the suggested concentration of this compound obtained in the body through a balanced diet, while the rutin concentration was chosen as the highest non-cytotoxic dose [14,28]. In parallel, cells were cultured without irradiation in medium containing the above supplements. In order to maintain the same conditions for all experimental and control groups, all media contained 0.1% DMSO.

**Figure 2:**
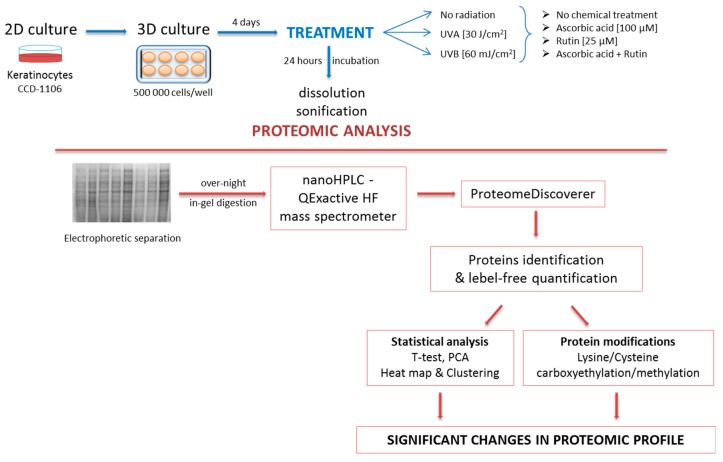
The scheme of the experiment including cells treatment, sample processing, and data statistical analysis.

Following incubation, keratinocytes were collected from 3D gel with AlgiMatrix™ dissolving buffer (Life Technologies, Carlsbad, CA, USA), lysed by sonification on ice, and centrifuged (15 min, 12,000× *g*). The total protein content in supernatant was measured using a Bradford assay [29]. Figure 2 shows the diagram of the experiment scheme.

### 2.2. Proteomic Analysis

The supernatant was mixed 1:1 with Laemmle buffer (supplemented with 5% 2-mercaptoethanol) and heated at 95 °C for 10 min for protein denaturation. Samples were separated on 10% Tris-Glycine SDS-PAGE gels and stained with Coomassie brilliant blue R-250. Complete lanes were cut out of the gel, sliced into 12 sections and in-gel digested overnight with trypsin (Promega, Madison, WI, USA). The resulting peptide mixture was extracted from the gel, dried, and dissolved in 5% ACN + 0.1% formic acid (FA) and separated using an Ultimate 3000 (Dionex, Idstein, Germany) onto a 150 mm × 75 mm PepMap RSLC capillary analytical C18 column (Dionex, LC Packings). The peptides eluted from the column were analyzed using a QExactive HF mass spectrometer with an electrospray ionization source (ESI) (Thermo Fisher Scientific, Bremen, Germany).

### 2.3. Protein Identification, Grouping, and Label-Free Quantification

Processing of the raw data generated from LC-MS/MS analysis was carried out using Proteome Discoverer 2.0 (Thermo Fisher Scientific, Bremen, Germany) and Sequest HT (SEQUEST HT algorithm, license Thermo Scientific, registered trademark University of Washington, USA). Input data were searched against the UniProtKB-SwissProt database (taxonomy: *Homo sapiens*, release 04/2018). For protein identification the following search parameters were used: peptide mass tolerance set to 10 ppm, MS/MS mass tolerance set to 0.02 Da, up to two missed cleavages allowed. Dynamic modifications of lysine or cysteine carboxyethylation (CEL/CEC) and carboxymethylation (CML/CMC) were set [30].

### 2.4. Statistical Analysis

Analysis of each sample were performed in three independent experiments. Results from individual protein label-free quantification were normalized by the sample sum, log transformed and analyzed using the standard statistical analysis methods, including T-test, principal component analysis (PCA), heat map and dendrogram creation with free available MetaboAnalyst 4.0 software (http://www.metaboanalyst.ca).

## 3. Results

**Figure 3:**
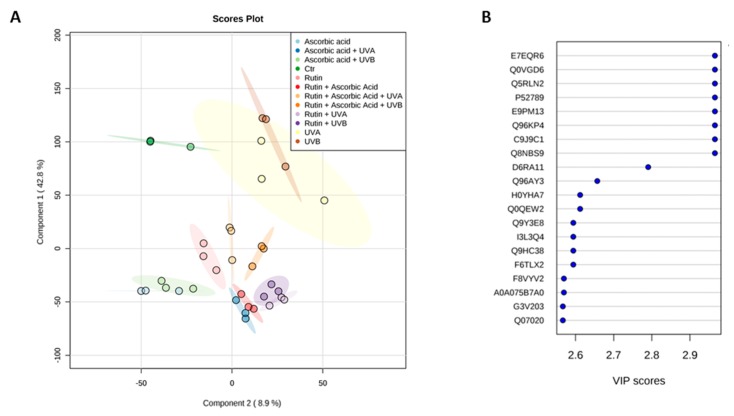
Principal component analysis (PCA) (**A**) and top 20 of component 1 VIP scores (**B**) for proteins from the 3D cultured keratinocytes exposed to UVA (30 J/cm^2^) or UVB irradiation (60 mJ/cm^2^) and treated with rutin (25 µM) or/and ascorbic acid (100 µM). Proteins: E7EQR6—T-complex protein 1 subunit α; Q0VGD6—heterogeneous nuclear ribonucleoprotein R protein; P52789—hexokinase-2; E9PM13—heat shock cognate 71 kDa protein; Q96KP4—cytosolic non-specific dipeptidase; C9J9C1—serine/threonine-protein phosphatase 2A; Q8NBS9—thioredoxin domain-containing protein 5; D6RA11—ubiquitin-conjugating enzyme E2 D3; Q96AY3—peptidyl-prolyl cis-trans isomerase; H0YHA7, Q0QEW2, F8VYV2, A0A075B7A0, G3V203, Q07020—ribosomal proteins; Q9Y3E8—CGI-150 protein; I3L3Q4, Q9HC38, F6TLX2—glyoxalase domain-containing proteins.

**Figure 4:**
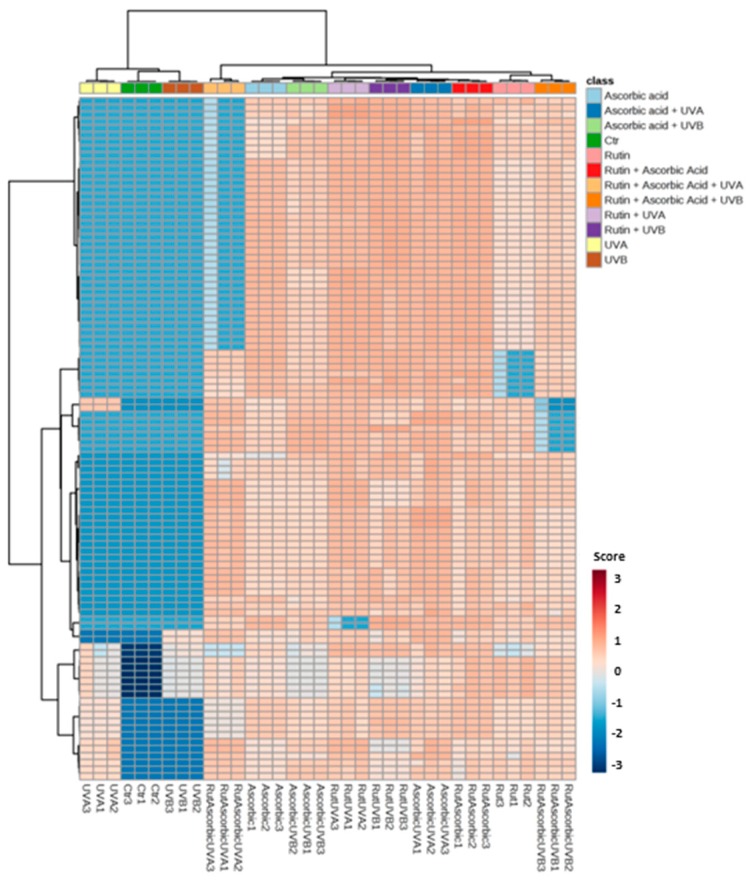
Heat map and clustering for the top 100 proteins from the 3D cultured keratinocytes exposed to UVA (30 J/cm^2^) or UVB irradiation (60 mJ/cm^2^) and treated with treated with rutin (25 µM) or/and ascorbic acid (100 µM). Protein expression levels (log transformed) were scaled to the row mean.

**Figure 5:**
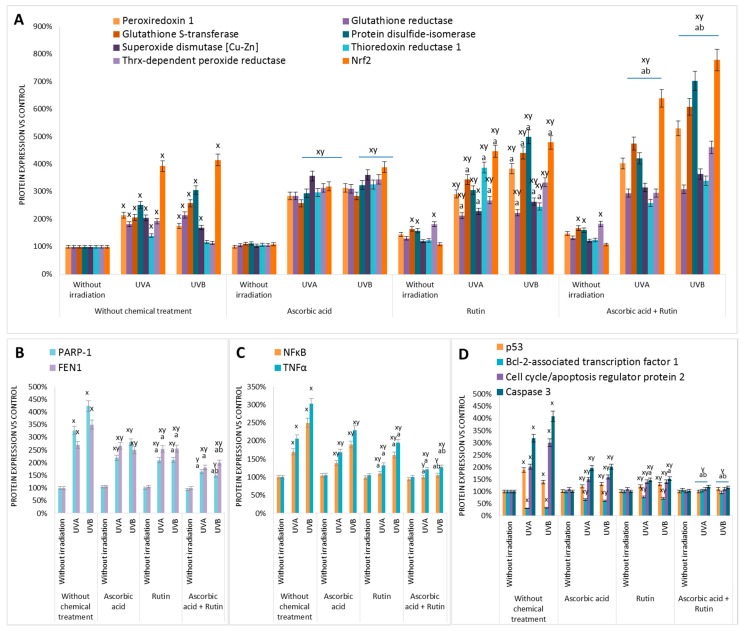
The expression of antioxidant (**A**), DNA repairing (**B**), proinflammatory (**C**), and pro/anti-apoptotic (**D**) proteins of which levels were changed in 3D cultured keratinocytes exposed to UVA (30 J/cm^2^) or UVB irradiation (60 mJ/cm^2^) and treated with treated with rutin (25 µM) or/and ascorbic acid (100 µM). Data obtained from label-free analysis. Mean values ± SD of three independent experiments are presented. ^x^ statistically significant differences vs. non-treated group, *p* < 0.05; ^y^ statistically significant differences vs. respectively group without chemical treatment, *p* < 0.05; ^a^ statistically significant differences vs. ascorbic acid treated group, *p* < 0.05; ^b^ statistically significant differences vs. rutin treated group, *p* < 0.05.

The results of this study showed that rutin and ascorbic acid treatments lead to significant changes in protein expression in UVA or UVB irradiated keratinocytes. For all samples 862 proteins with at least two unique peptides were identified and measured (Table S1). As is shown in Figure 3, there was a strong differentiation between all treatment conditions following principal component analysis (PCA) (component 1–42.8%; component 2–8.9%). The top 20 proteins from PCA component 1, along with their VIP scores, are presented in Figure 2. Moreover, these results were confirmed by the hierarchical clustering of the top 100 proteins with a significant *p*-value (Figure 4). Comparing the *p*-values allowed us to identify which proteins had the highest differences in expression following rutin and ascorbic acid treatment (Figure 5). The main changes observed were in the case of proteins involved in antioxidant response, including peroxiredoxin 1, glutathione reductase, glutathione S-transferase, protein disulfide-isomerase, superoxide dismutase (Cu–Zn), thioredoxin reductase 1, thioredoxin-dependent peroxide reductase, and the cytoprotective transcription factor, Nrf2. The expression of these proteins changed following rutin and ascorbic acid treatment, which counteracted UV-induced alterations. In addition, the UV induced increase in DNA repairing proteins level, such as PARP-1 and FEN1 (Poly(ADP–ribose) polymerase 1, Flap endonuclease 1), was partially decreased by the protective rutin and ascorbic acid action. Simultaneously, rutin and ascorbic acid decreased the expression of some proinflammatory (NFκB, TNFα) and proapoptotic (p53, cell cycle/apoptosis regulator protein 2, caspase 3) proteins. In all, cases rutin treatment, following UV radiation, showed slightly stronger cytoprotective effects than ascorbic acid, however, the use of these two antioxidants in the cases of proapoptotic and proinflammatory proteins allowed the UV irradiated cells to restore the level of mentioned proteins following cell stress.

**Figure 6:**
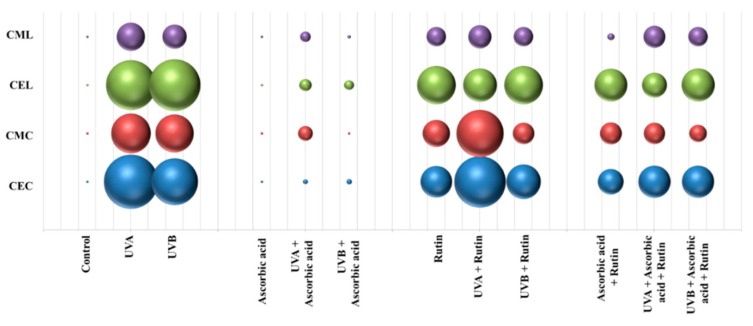
The comparison of the lysine or cysteine modifications (carboxyethylation (CEL/CEC) and carboxymethylation (CML/CMC)) level in 3D cultured keratinocytes exposed to UVA (30 J/cm^2^) or UVB irradiation (60 mJ/cm^2^) and treated with treated with rutin (25 µM) or/and ascorbic acid (100 µM). Data obtained from label-free analysis.

UV-induced oxidative stress also significantly increased post-translational protein modifications (PTMs), including lysine/cysteine carboxyethylation and carboxymethylation (Figure 6). While ascorbic acid significantly reduced the level of these modifications, rutin favored their creation under both standard conditions and following UV-induced stress. However, combined use of rutin and ascorbic acid decreased the level of analyzed PTMs in keratinocytes exposed to UV radiations.

## 4. Discussion

Permanent exposure of skin cells to solar radiation induces disturbances in cell metabolism via UV radiation. This creates the need for research and development of natural methods of protection against harmful effects of UV radiation. UV-induced modifications of both bioactive and structural proteins are some of the most dangerous changes in the cellular proteome. In order to maximize the effective protection of skin cells, single treatment therapies are now being replaced by combination therapies [14]. In addition, the testing of harmful effects of UV radiation and cytoprotection of selected compounds in the traditional two-dimensional (2D) culture system does not allow full understanding of the cellular phenotype and cell–cell interaction [31]. Therefore, we assessed the combined effect of ascorbic acid and rutin on the proteomic profile in keratinocytes grown in a three-dimensional (3D) system in an attempt to replicate the skin microenvironment.

This study combined the use of rutin and ascorbic acid, widely known as oral pharmacological agents for the prevention of flu and colds. However, such formulations, despite cytoprotective properties, are not commonly used together in preparations for skin care and protection. While ascorbic acid is frequently added to topical ointments, its interaction with rutin is still unknown apart from the fact that ascorbic acid enhances membranes penetration by rutin [21]. Moreover, water soluble ascorbic acid, as well as high molecular rutin have low absorption into the skin, which can be additionally compensated by oral supplementation. Literature data shows that oral supplementation of rutin in a standard dose 500 mg/day induces a 100 mg/L concentration of this molecule in the plasma following 5 h after ingestion, and decreases in the next five hours to 10 mg/L [32], which are comparable to the conditions used in this study. 

Both of the compounds used in this study, have exhibited in previous studies selective anti-inflammatory effects in UV-irradiated cells. It has been shown that ascorbic acid is able to downregulate IL-1β mRNA expression in UVA-irradiated and IL-8 mRNA in UVB-irradiated keratinocytes [33,34]. Additionally, rutin may influence UV-induced proinflammatory signaling via inhibition of the enzymes responsible for metabolism of fatty acids and reducing the generation of proinflammatory signaling molecules [10,16]. However, the combined action of rutin and ascorbic acid, as observed in this study and previous data from 2D culture keratinocytes [14], led to a reduction in the biological activity of NFκB, resulting in a significant reduction in TNFα levels. Therefore, the functional activity of rutin and ascorbic acid in UV irradiated keratinocytes leads to inhibition of inflammatory processes. Presented proteomic data are consistent with previous data obtained by Western blot analysis for 2D cultured keratinocytes [14].

UV radiation induced inflammation was partially reduced by the combined action of rutin and ascorbic acid. Both rutin and ascorbic acid support the cellular antioxidant system, not only as scavengers of free radicals, but also by stimulating the biosynthesis of antioxidant proteins. Data obtained in this study showed that the most UV-sensitive proteins were combined with a glutathione-based system (GSH) (glutathione reductase, glutathione S-transferase) or thioredoxin-based system (thioredoxin reductase, peroxiredoxin, thioredoxin-dependent peroxide reductase, protein disulfide-isomerase). GSH is a cofactor for GSH peroxidase, which is also responsible for protecting lipids from peroxidation [35]. Therefore, the appropriate level of GSH, delivered by the high activity of glutathione reductase and glutathione S-transferase, supports the antioxidant system of keratinocytes during oxidative stress. Alternatively, the thioredoxin-dependent antioxidant system can be activated under oxidative conditions, which is particularly important in the repair of UV-induced protein modifications [36]. However, it has also been shown that many antioxidant compounds with the ability to thioredoxin associated activate enzymes can act as cytoprotectors against UV radiation in keratinocytes [37,38,39].

The reaction of keratinocytes to UV-induced oxidative stress is also associated with the activation of intracellular and extracellular signaling pathways. These modifications can lead to changes in Nrf2 transcription factor activity. Nrf2 is a transcription factor bound to the cytoplasm by Keap1 that is responsible for the expression of genes encoding antioxidant and anti-apoptotic proteins [40,41]. This reaction was further intensified by rutin and ascorbic acid, especially when used in combination, which prepared the cells for oxidative stress [42]. Similar data have been presented previously for the 2D cultured keratinocytes, where rutin and ascorbic acid additionally increased Nrf2 level also by enhancing its activator KAP1 and decreasing nuclear inhibitor Bach1 [14]. On the other hand, rutin, by forming adducts with Keap1, changes the conformation of this molecule and additionally leads to dissociation of the Keap1–Nrf2 complex [15].

The mentioned antioxidant activity of ascorbic acid and rutin may also be an explanation for the reduction in DNA repairing protein expression following cells exposure to UVA and UVB radiation. It is known that UV irradiation causes direct nucleic acid damage or leads to indirect genetic material destruction also by ROS dependent DNA oxidation [43]. These disturbances induce the DNA repairing system that also includes an increase in the expression of protein involved in DNA repair [6]. Examples of such proteins are PARP-1 and FEN1, of which increased expression following cell UV irradiation has been found previously [44,45]. However, because of the activation of the antioxidant system and reduction of the oxidative UV radiation potential, ascorbic acid and rutin partially prevented the UV-induced increase in the expression of these proteins.

UV radiation induced apoptosis also reduces cell viability [46]. This has been confirmed as associated with increased levels of pro-apoptotic proteins, such as cytochrome c or caspase 3 [47]. As shown in this study, combination of rutin and ascorbic acid protects keratinocytes from UV-induced increased expression of caspase 3, which was previously confirmed by Western blot analysis for 2D cultured keratinocytes [14]. As of now, ascorbic acid has been shown to modify the process of apoptosis. It also has been shown to stimulate apoptosis in cancer cells [48] and prevent apoptosis of unchanged cancer cells subjected to a single stress signal [49]. In the case of keratinocytes, ascorbic acid is important for the initiation of keratinization, and thus is also required for cell survival and differentiation [50]. However, rutin is known as an inhibitor of caspase 9, and may be linked to a lack of caspase 3 activation [51]. This was observed in the present study as a decreased expression of the protein. Similar effects of rutin have been previously observed in the case of protein p53 in the mouse kidney [52]. However, in the case of UV irradiated keratinocytes, decreased levels of p53 and cell cycle/apoptosis proteins prevented cell cycle arrest and promoted proper keratinization and natural keratosis of the epidermis that previously was not visible in the case of cells cultured in monolayer [14].

As is shown in the PCA analysis, the proteins most sensitive to UV radiation and rutin/ascorbic acid treatment were also the molecules involved in the protein biosynthesis, including ribonucleoproteins and ribosomal proteins, as well as molecules involved in protein folding and maturation (heat shock proteins, chaperon: T-complex protein (1)). The data presented here show that rutin alone was able to restore the level of these proteins after stress more than ascorbic acid alone, in agreement with previous work [53]. Another important cell metabolism protein affected by UV radiation, and protected by use of antioxidants, is serine/threonine-protein phosphatase 2A (PP2A), a major phosphatase for microtubule-associated proteins [54]. PP2A modulates the activity of a number of kinases (mitogen-stimulated S6 kinase, MAP-2 kinase, casein kinase 2, or Raf1) and provides cells with fluent signal transmission [55]. Moreover, the combined effect of rutin and ascorbic acid, restores PP2A levels in UV irradiated keratinocytes and may protect cells against apoptosis via PP2A dependent de-phosphorylation of p53 [56]. Further, UV induced changes in expression of glyoxalase domain-containing proteins may lead to disturbances in intracellular cadherin binding and inhibition of cell growth [57]. Therefore, the rutin and ascorbic acid induced decrease in protein levels contributed to the protection of UV-irradiated keratinocytes against apoptosis.

Alternatively, rutin and ascorbic acid can also influence glucose metabolism by modifying hexokinase 2 levels. While ascorbic acid restored the UV-enhanced level of this enzyme, rutin significantly increased hexokinase 2 levels in both control and UV irradiated keratinocytes. Hexokinase phosphorylates glucose to produce glucose-6-phosphate (G6P), which is the first step in energetic metabolism [58]. Flavonoids, including rutin are known to stimulate this process [59]. As a result of increased sugar metabolism, the enhanced levels of AGEs are observed [60]. On the other hand, medium supplemented with rutin was characterized by enhanced pH that additionally favors protein carboxymethylation/carboxyethylation [61]. Therefore, rutin may be a stimulator of this process. Such modifications lead to interruption of protein–protein interactions, polysaccharide–protein complex formation that are considered markers of arteriosclerosis, diabetes mellitus, and aging [62], and also have been shown to operate as a potent antitumor factors [63]. As a consequence, rutin may affect intracellular signaling pathways. However, rutin combined with ascorbic acid partially reduced this effect.

The results shown here indicate that rutin and ascorbic acid, could modify UV induced dysregulation of cellular metabolism. However, the combination of rutin and ascorbic acid showed a stronger cytoprotective effect than when using either of compound alone. These results suggest that this synergistic effect may result from mutual support of penetration through biological membranes. Therefore, the cooperation of rutin and ascorbic acid in the cytoprotective effect on keratinocytes exposed to UVA and UVB radiation makes them a potentially effective protective system against skin damage caused by UV radiation.

## Supplementary Materials

The following are available online at https://www.mdpi.com/2072-6643/11/11/2672/s1, Table S1: The list of proteins with at least 2 unique peptides identified in 3D cultured keratinocytes exposed to UVA (30 J/cm^2^) or UVB irradiation (60 mJ/cm^2^) and treated with treated with rutin (25 µM) or/and ascorbic acid (100 µM).
