# Supplementary material for: Synergistic Cytoprotective Effects of Rutin and Ascorbic Acid on the Proteomic Profile of 3D-Cultured Keratinocytes Exposed to UVA or UVB Radiation
Source: Nutrients. 2019 Nov 5;11(11):2672. doi: 10.3390/nu11112672 (PMC6893536; doi:10.3390/nu11112672)
Supplement: Supplementary file 1 [file nutrients-11-02672-s001.pdf]

Supplementary table 1. The list of proteins with at least 2 unique peptides identified in 3D cultured keratinocytes exposed to UVA (30 J/cm^2) or UVB irradiation (60 mJ/cm^2) and treated with treated with rutin [25 µM] or/and ascorbic acid [100 µM].

Nr	**Accession**	**Description**
1	A0A024QZN4	Vinculin
2	A0A024QZN9	Voltage-dependent anion channel 2
3	A0A024QZV0	HCG1811539
4	A0A024QZX3	Serpin peptidase inhibitor
5	A0A024QZZ7	Histone H2B
6	A0A024R1A3	Ubiquitin-activating enzyme E1
7	A0A024R1K7	Tyrosine 3-monooxygenase/tryptophan 5-monooxygenase activation protein
8	A0A024R280	Phosphoserine aminotransferase 1
9	A0A024R2Q4	Ribosomal protein L15
10	A0A024R321	Filamin B
11	A0A024R382	CNDP dipeptidase 2
12	A0A024R3V9	HCG37498
13	A0A024R3X7	Heat shock 10kDa protein 1 (Chaperonin 10)
14	A0A024R408	Actin related protein 2/3 complex, subunit 2,
15	A0A024R4U3	Tubulin tyrosine ligase-like family
16	A0A024R592	Glucosidase
17	A0A024R5Z8	RAB11A, member RAS oncogene family
18	A0A024R652	Methylenetetrahydrofolate dehydrogenase
19	A0A024R6C9	Dihydrolipoamide S-succinyltransferase
20	A0A024R6D4	Enhancer of rudimentary homolog
21	A0A024R7F7	Transportin 2
22	A0A024R7T3	Heterogeneous nuclear ribonucleoprotein F
23	A0A024R814	Ribosomal protein L7
24	A0A024R872	Chromosome 9 open reading frame 88
25	A0A024R895	SET translocation
26	A0A024R8W0	DEAD (Asp-Glu-Ala-Asp) box polypeptide 48
27	A0A024R9E2	Poly(A) binding protein, cytoplasmic 1
28	A0A024RA28	Heterogeneous nuclear ribonucleoprotein A2/B1
29	A0A024RA52	Proteasome subunit alpha
30	A0A024RAE4	Cell division cycle 42
31	A0A024RAM0	Transportin 1
32	A0A024RAM2	Glutaredoxin (Thioltransferase)
33	A0A024RB41	HCG2016482
34	A0A024RB85	Proliferation-associated 2G4
35	A0A024RBH2	Cytoskeleton-associated protein 4
36	A0A024RBK9	Thioredoxin reductase 1
37	A0A024RC46	HCG2020860
38	A0A024RC76	Interferon stimulated exonuclease gene 20kDa
39	A0A024RC87	Ribonuclease/angiogenin inhibitor 1

40	A0A024RCN6	Valyl-tRNA synthetase
41	A0A024RD93	Phosphoribosylaminoimidazole carboxylase
42	A0A024RDH6	SEC31-like 1
43	A0A024RDQ0	Heat shock 105kDa/110kDa protein 1
44	A0A075B6E2	40S ribosomal protein S19
45	A0A075B7A0	Ribosomal protein L5
46	A0A087WTP3	Far upstream element-binding protein 2
47	A0A087WUV8	Basigin
48	A0A087WUZ3	Spectrin beta chain, non-erythrocytic 1
49	A0A087WVQ9	Elongation factor 1-alpha 1
50	A0A087WW66	26S proteasome non-ATPase regulatory subunit 1
51	A0A087WX41	Clathrin heavy chain 2
52	A0A087WYC1	Heat shock 70 kDa protein 4
53	A0A087WYG8	Alpha-internexin
54	A0A087WYS1	UTP--glucose-1-phosphate uridylyltransferase
55	A0A087WZ27	Zinc finger protein 90
56	A0A087X0X3	Heterogeneous nuclear ribonucleoprotein M
57	A0A087X1N8	Serpin B6
58	A0A087X1Z3	Proteasome activator complex subunit
59	A0A087X253	AP-2 complex subunit beta
60	A0A087X2E9	Glutathione S-transferase P
61	A0A090N8G0	Glycyl-tRNA synthetase
62	A0A0A0MSA7	Eukaryotic translation initiation factor 4 gamma 3
63	A0A0A0MSI0	Peroxiredoxin 1
64	A0A0A0MSS8	Aldo-keto reductase family 1 member C3
65	A0A0A0MTN3	Glutathione S-transferase Mu
66	A0A0A0U6N4	NADP+-soluble isocitrate dehydrogenase 1
67	A0A0A6YYG9	Protein ARPC4-TTLL3
68	A0A0B4J207	Ribose-phosphate pyrophosphokinase 3
69	A0A0C4DFS8	Nicotinamide phosphoribosyltransferase
70	A0A0C4DFV9	Protein SET
71	A0A0C4DG17	40S ribosomal protein SA
72	A0A0C4DGH5	Cullin-associated NEDD8-dissociated protein 1
73	A0A0D9SFB3	ATP-dependent RNA helicase DDX3X
74	A0A0D9SFF6	Spectrin alpha chain, non-erythrocytic 1
75	A0A0G2JJZ9	Spliceosome RNA helicase DDX39B
76	A0A0K0K1H9	Epididymis secretory protein Li 48
77	A0A0K0K1I0	Epididymis secretory protein Li 265
78	A0A0S2Z3C0	Actinin alpha 4 isoform 3
79	A0A0S2Z3W2	Fumarate hydratase isoform 6
80	A0A0S2Z3X3	Actinin alpha 4 isoform 2
81	A0A0S2Z3X6	Fumarate hydratase isoform 4
82	A0A0S2Z3Y1	Lectin galactoside-binding soluble 3 binding protein
83	A0A0S2Z415	Heat shock 60kDa protein 1
84	A0A0S2Z428	HCG2039812

85	A0A0S2Z434	Hydroxysteroid dehydrogenase 10
86	A0A0S2Z471	Creatine kinase brain isoform 2
87	A0A0S2Z4G4	Tropomyosin 3 isoform 1
88	A0A0S2Z4G8	Tropomyosin 3 isoform 2
89	A0A0S2Z4H0	Fumarate hydratase isoform 5
90	A0A0S2Z4I4	Tropomyosin 3 isoform 3
91	A0A0S2Z4J7	Glutathione synthetase isoform 2
92	A0A0S2Z5K8	Spermatid perinuclear RNA binding protein isoform 2
93	A0A0U1RQF0	Fatty acid synthase
94	A0AUL6	ACTB protein
95	A0JLQ5	BXDC2 protein
96	A0PJ87	RSL1D1 protein
97	A1JUI8	Chaperonin subunit 6A
98	A1L0V1	ACTN1 protein
99	A1XP52	Catecholamine-regulated protein 40
100	A2IDB2	14-3-3 protein eta
101	A3R0T8	Histone 1
102	A4D2P0	Ras-related C3 botulinum toxin substrate 1
103	A4FU77	SNRNP200 protein
104	A4FU99	CLTCL1 protein
105	A4QMW8	ENO1 protein
106	A4QPB0	IQ motif containing GTPase activating protein 1
107	A4UCS5	Peroxiredoxin 3
108	A4UCS6	Peroxiredoxin 6
109	A4UCS8	Enolase 1
110	A4UCT3	Beta-actin
111	A4UCU2	Beta-4 tubulin
112	A4ZU86	Truncated nucleolar phosphoprotein B23
113	A5D904	RPS9 protein
114	A5YM63	NEFM protein
115	A6NIW5	Peroxiredoxin 2
116	A6NJA2	Ubiquitin carboxyl-terminal hydrolase 14
117	A6NLN1	Polypyrimidine tract binding protein 1
118	A6XGP7	Reticulon
119	A7E2S3	RPS3 protein
120	A7XZE4	Beta tropomyosin
121	A8K168	Malic enzyme
122	A8K7Q2	Heat shock cognate 71 kDa protein
123	A8K7T0	Kynureninase
124	A8K8N7	Phosphoribosylformylglycinamidine synthase
125	A9X7H1	Eukaryotic translation elongation factor 1 alpha
126	B0YJC4	Vimentin
127	B1AHC9	X-ray repair cross-complementing protein 6
128	B1AP13	Complement decay-accelerating factor
129	B1AP15	CD55 antigen

130	B1AQP8	Epoxide hydrolase 1
131	B2MV14	Truncated lactoferrin
132	B2R4D8	60S ribosomal protein L27
133	B2RPK0	Putative high mobility group protein B1
134	B2RU10	EIF4G1 protein
135	B2RXH8	Heterogeneous nuclear ribonucleoprotein C-like 2
136	B2ZZ89	Epididymis luminal protein 102
137	B3KRS7	RuvB-like helicase
138	B3KS31	Tubulin, beta 6
139	B3KWE1	Histidine--tRNA ligase, cytoplasmic
140	B4DID5	Transaldolase 1
141	B4DJV2	Citrate synthase
142	B4DK69	Aldo-keto reductase family 1 member C2
143	B4DLV8	Ribosomal protein L9
144	B4DMJ7	HCG2015269
145	B4DP52	HCG2005638
146	B4DT28	Heterogeneous nuclear ribonucleoprotein R
147	B4DUR8	T-complex protein 1 subunit gamma
148	B4DVZ8	Leukotriene A(4) hydrolase
149	B4DXW1	Actin-related protein 3
150	B4E0E1	Poly [ADP-ribose] polymerase
151	B5BU24	14-3-3 protein beta/alpha
152	B5BU25	U2 small nuclear RNA auxiliary factor 2
153	B5BUE6	ATP-dependent RNA helicase DDX5
154	B5MCE7	Basic leucine zipper and W2 domain-containing protein 2
155	B5MCP9	40S ribosomal protein S7
156	B5MCT8	40S ribosomal protein S9
157	B5MDF5	GTP-binding nuclear protein Ran
158	B7Z268	Single-stranded DNA binding protein 1
159	B7Z2E6	14-3-3 protein zeta/delta
160	B7Z4E3	60S ribosomal protein L31
161	B7Z645	Heterogeneous nuclear ribonucleoprotein Q
162	B7ZB02	Ribose-phosphate pyrophosphokinase 1
163	B8Y0L3	Aspartate beta-hydroxylase
164	B8ZZ38	Leucine-rich PPR motif-containing protein, mitochondrial
165	B8ZZL8	10 kDa heat shock protein, mitochondrial
166	B9EKV4	Aldehyde dehydrogenase 9 family, member A1
167	C9J0D1	Histone H2A
168	C9J4N6	Isocitrate dehydrogenase [NADP] cytoplasmic
169	C9J712	Profilin-2
170	C9J8F3	Fructose-bisphosphate aldolase C
171	C9J9C1	Serine/threonine-protein phosphatase 2A
172	C9JFN4	Basic leucine zipper and W2 domain-containing protein 1
173	C9JFR7	Cytochrome c
174	C9JMZ3	Aminopeptidase B

175	C9JRZ8	Aldo-keto reductase family 1 member B15
176	C9JUP7	Transitional endoplasmic reticulum ATPase
177	C9JZG2	Trifunctional purine biosynthetic protein adenosine-3
178	C9K025	60S ribosomal protein L35a
179	C9K0U8	Single-stranded DNA-binding protein, mitochondrial
180	D3DN77	HCG2022736
181	D3DP78	Aspartyl-tRNA synthetase
182	D3DPI2	HCG1641229
183	D3DQY9	HCG1991922
184	D3DS95	HCG21173
185	D3DTL4	Enolase 3
186	D3DUG9	Ubiquitin specific peptidase 14
187	D3DWK1	Eukaryotic translation elongation factor 1 delta
188	D6R9B6	40S ribosomal protein S3a
189	D6R9P3	Heterogeneous nuclear ribonucleoprotein A/B
190	D6RA11	Ubiquitin-conjugating enzyme E2
191	D6RBW1	Eukaryotic translation initiation factor 4E
192	D6RC06	Histidine triad nucleotide-binding protein 1
193	D6RCN3	Annexin A5
194	D6REM6	Matrin-3
195	D6RF62	Multifunctional protein ADE2
196	D6W507	HCG1990625
197	D6W5E6	UDP-glucose pyrophosphorylase 2
198	D9IAI1	Epididymis secretory protein Li 34
199	D9YZV4	Tropomyosin 1
200	E2DRY6	C-myc promoter-binding protein 1
201	E4W6B6	RPL27/NME2 fusion protein
202	E5RI06	Glutathione reductase, mitochondrial
203	E5RJH3	60S ribosomal protein L30
204	E7ENZ3	T-complex protein 1 subunit epsilon
205	E7EPB3	60S ribosomal protein L14
206	E7EQR6	T-complex protein 1 subunit α
207	E7ER44	Lactotransferrin
208	E7ESE0	60S ribosomal protein L9
209	E7ET17	Peroxisomal multifunctional enzyme type 2
210	E7ETK0	40S ribosomal protein S24
211	E7EX73	Eukaryotic translation initiation factor 4 gamma 1
212	E7EXB4	60 kDa heat shock protein, mitochondrial
213	E9PCX2	Aldose reductase
214	E9PCY7	Heterogeneous nuclear ribonucleoprotein H
215	E9PEB5	Far upstream element-binding protein 1
216	E9PEX6	Dihydrolipoyl dehydrogenase
217	E9PF18	Hydroxyacyl-coenzyme A dehydrogenase, mitochondrial
218	E9PFG7	2-oxoglutarate dehydrogenase, mitochondrial
219	E9PG15	14-3-3 protein theta

220	E9PGM4	1,4-alpha-glucan-branching enzyme
221	E9PGT1	Translin
222	E9PK47	Alpha-1,4 glucan phosphorylase
223	E9PKZ0	60S ribosomal protein L8
224	E9PLK3	Puromycin-sensitive aminopeptidase
225	E9PM13	Heat shock cognate 71 kDa protein
226	E9PM36	40S ribosomal protein S2
227	E9PM69	26S protease regulatory subunit 6A
228	E9PMA9	Ribonuclease inhibitor
229	E9PP73	Coatomer subunit beta
230	E9PPH5	Acidic leucine-rich nuclear phosphoprotein 32 family member E
231	E9PQ63	Carbonyl reductase [NADPH] 1
232	E9PRY8	Elongation factor 1-delta
233	E9PSF4	40S ribosomal protein S3
234	F1T0B3	ATP-dependent RNA helicase DDX1
235	F4ZW62	NF45
236	F4ZW64	NF90a
237	F4ZW66	NF110b
238	F5GXD8	Stress-induced-phosphoprotein 1
239	F5GZS6	4F2 cell-surface antigen heavy chain
240	F5H0C8	Gamma-enolase
241	F5H2F4	C-1-tetrahydrofolate synthase, cytoplasmic
242	F5H365	Protein transport protein Sec23A
243	F5H6X6	Neutral alpha-glucosidase AB
244	F5H8H6	L-lactate dehydrogenase
245	F6TLX2	Glyoxalase domain-containing protein 3
246	F6XY72	HCG2001850
247	F8VPD4	CAD protein
248	F8VQ14	T-complex protein 1 subunit beta
249	F8VS02	Alpha-aminoadipic semialdehyde dehydrogenase
250	F8VU65	60S acidic ribosomal protein P0
251	F8VX68	Citrate synthase, mitochondrial
252	F8VY02	Endoplasmic reticulum resident protein 29
253	F8VYV2	60S ribosomal protein L18
254	F8VZ45	60S ribosomal protein L6
255	F8W1N5	Nascent polypeptide-associated complex
256	F8W727	60S ribosomal protein L32
257	F8WD96	Cathepsin D
258	G3V1A4	Cofilin 1 (Non-muscle)
259	G3V1B3	60S ribosomal protein L21
260	G3V1C3	Apoptosis inhibitor 5
261	G3V203	Ribosomal protein S2
262	G3V213	Adenylate kinase 2
263	G3V2J8	Heat shock protein HSP 90-alpha
264	G3V576	Heterogeneous nuclear ribonucleoproteins C1/C2

265	G3V5B3	ERO1-like protein alpha
266	G3V5L0	Serine hydroxymethyltransferase, mitochondrial
267	G3V5Q1	DNA-(apurinic or apyrimidinic site) lyase
268	G3XAL0	Malate dehydrogenase
269	G5E9G0	60S ribosomal protein L3
270	G5E9R0	Actin, cytoplasmic 1
271	G5E9S2	Hydroxysteroid (17-beta) dehydrogenase 4
272	G9K388	YWHAE/FAM22A fusion protein
273	H0UID5	AP complex subunit beta
274	H0Y426	Valine--tRNA ligase
275	H0Y4R1	Inosine-5'-monophosphate dehydrogenase 2
276	H0YDZ7	Guanine deaminase
277	H0YFD6	Trifunctional enzyme subunit alpha, mitochondrial
278	H0YHA7	Ribosomal protein L10
279	H0YIZ0	Serine hydroxymethyltransferase
280	H0YJ63	Activator of 90 kDa heat shock protein ATPase homolog 1
281	H0YJC0	26S protease regulatory subunit 10B
282	H0YK49	Electron transfer flavoprotein subunit alpha, mitochondrial
283	H0YKD8	60S ribosomal protein L28
284	H0YKN4	Annexin A2
285	H0YL72	Isocitrate dehydrogenase [NAD] subunit alpha
286	H0YLC7	Fumarylacetoacetase
287	H0YLE8	Ras GTPase-activating-like protein IQGAP1
288	H0YN26	Acidic leucine-rich nuclear phosphoprotein 32 family member A
289	H0YN88	40S ribosomal protein S17
290	H3BM89	60S ribosomal protein L4
291	H3BNI9	Casein kinase II subunit alpha
292	H3BNT7	26S proteasome non-ATPase regulatory subunit 7
293	H3BQZ9	Adenine phosphoribosyltransferase
294	H3BRV9	Nuclear transport factor 2
295	H3BSC1	Ras-related protein Rab
296	H3BU13	Pyruvate kinase PKM
297	H6WCP5	EPRSN1
298	H7BZ35	Aspartate--tRNA ligase, cytoplasmic
299	H7C004	Serpin B10
300	H7C0R3	Glutamine--tRNA ligase
301	H7C123	60S ribosomal protein L10
302	H7C1H2	26S proteasome non-ATPase regulatory subunit 2
303	H7C3F9	Actin-related protein 2/3 complex subunit 2
304	H7C3T4	Peroxiredoxin 4
305	H9ZYJ2	Thioredoxin
306	I3L0A0	HCG2044781
307	I3L239	Heat shock protein 75 kDa, mitochondrial
308	I3L2R6	Thioredoxin domain-containing protein 17
309	I3L3P7	40S ribosomal protein S15a

310	I3L3Q4	Glyoxalase domain-containing protein 4
311	I6L957	HNRNPA2B1 protein
312	I6L965	KRT18 protein
313	I6TRR8	SND1-BRAF
314	J3JS69	40S ribosomal protein S18
315	J3K000	PEPD protein
316	J3KMX5	40S ribosomal protein S13
317	J3KNQ3	26S proteasome non-ATPase regulatory subunit 13
318	J3KPD9	Protein NME1-NME2
319	J3KPE3	Receptor of-activated protein C kinase 1
320	J3KQ18	D-dopachrome decarboxylase
321	J3KQ32	Obg-like ATPase 1
322	J3KRD5	Cytosolic non-specific dipeptidase
323	J3KRY1	Rho GDP-dissociation inhibitor 1
324	J3KS13	Clathrin heavy chain 1
325	J3QR09	Ribosomal protein L19
326	J3QR64	Eukaryotic initiation factor 4A-I
327	J3QTR3	Ubiquitin-40S ribosomal protein S27a
328	J7M2B1	Tyrosine-protein kinase receptor
329	K7EJ44	Profilin 1
330	K7EKS7	60S ribosomal protein L22
331	K7ELC2	40S ribosomal protein S15
332	K7ELW0	Protein deglycase DJ-1
333	K7EM49	6-phosphogluconate dehydrogenase, decarboxylating
334	K7ENG2	Splicing factor U2AF 65 kDa
335	K7EQ48	Glucose-6-phosphate isomerase
336	K7ER00	Phenylalanine--tRNA ligase alpha subunit
337	K9MS24	Beta-spectrin non-erythrocytic 1
338	L0R849	Alternative protein EDARADD
339	L8B4R0	Ubiquitin C
340	M0QZC5	40S ribosomal protein S11
341	M0R0R2	40S ribosomal protein S5
342	M0R192	Flavin reductase (NADPH)
343	M0R1A7	60S ribosomal protein L18a
344	M0R1M6	Ubiquitin-60S ribosomal protein L40
345	M0R210	40S ribosomal protein S16
346	M0R248	Delta(3,5)-Delta(2,4)-dienoyl-CoA isomerase, mitochondrial
347	M0R261	6-phosphogluconolactonase
348	O00231	26S proteasome non-ATPase regulatory subunit 11
349	O00303	Eukaryotic translation initiation factor 3 subunit F
350	O00487	26S proteasome non-ATPase regulatory subunit 14
351	O14744	Protein arginine N-methyltransferase 5
352	O14942	Heat shock protein beta
353	O14943	Phosphofructokinase-P
354	O14980	Exportin-1

355	O14992	HS24/P52
356	O43143	Pre-mRNA-splicing factor ATP-dependent RNA helicase
357	O43242	26S proteasome non-ATPase regulatory subunit 3
358	O43707	Alpha-actinin-4
359	O60218	Aldo-keto reductase family 1 member B10
360	O60568	Procollagen-lysine,2-oxoglutarate 5-dioxygenase 3
361	O60701	UDP-glucose 6-dehydrogenase
362	O60812	Heterogeneous nuclear ribonucleoprotein C-like 1
363	O75322	Hsp89-alpha-delta-N
364	O75367	Core histone macro-H2A.1
365	O75533	Splicing factor 3B subunit 1
366	O75643	U5 small nuclear ribonucleoprotein 200 kDa helicase
367	O75828	Carbonyl reductase [NADPH] 3
368	O76003	Glutaredoxin-3
369	O76021	Ribosomal L1 domain-containing protein 1
370	O95758	Polypyrimidine tract-binding protein 3
371	P00491	Purine nucleoside phosphorylase
372	P00492	Hypoxanthine-guanine phosphoribosyltransferase
373	P00966	Argininosuccinate synthase
374	P01375	TNFα
375	P02765	Alpha-2-HS-glycoprotein
376	P02771	Alpha-fetoprotein
377	P02792	Ferritin light chain
378	P02794	Ferritin heavy chain
379	P04075	Fructose-bisphosphate aldolase A
380	P04083	Annexin A1
381	P04179	Superoxide dismutase [Mn], mitochondrial
382	P04632	Calpain small subunit 1
383	P04637	P53
384	P05091	Aldehyde dehydrogenase, mitochondrial
385	P05198	Eukaryotic translation initiation factor 2 subunit 1
386	P06733	Alpha-enolase
387	P07954	Fumarate hydratase, mitochondrial
388	P08238	Heat shock protein HSP 90-beta
389	P09382	Galectin-1
390	P0CG47	Polyubiquitin-B
391	P0DMR1	Heterogeneous nuclear ribonucleoprotein C-like 4
392	P0DMV9	Heat shock 70 kDa protein 1B
393	P10155	60 kDa SS-A/Ro ribonucleoprotein
394	P11279	Lysosome-associated membrane glycoprotein 1
395	P11413	Glucose-6-phosphate 1-dehydrogenase
396	P11908	Ribose-phosphate pyrophosphokinase 2
397	P11940	Polyadenylate-binding protein 1
398	P12814	Alpha-actinin-1
399	P13010	X-ray repair cross-complementing protein 5

400	P13639	Elongation factor 2
401	P13693	Translationally-controlled tumor protein
402	P13929	Beta-enolase
403	P14324	Farnesyl pyrophosphate synthase
404	P14550	Alcohol dehydrogenase [NADP(+)]
405	P14625	Endoplasmin
406	P14678	Small nuclear ribonucleoprotein-associated proteins B
407	P14866	Heterogeneous nuclear ribonucleoprotein L
408	P15170	Eukaryotic peptide chain release factor GTP-binding subunit ERF3A
409	P15559	NAD(P)H dehydrogenase [quinone] 1
410	P16402	Histone H1.3
411	P16403	Histone H1.2
412	P17066	Heat shock 70 kDa protein 6
413	P17655	Calpain-2 catalytic subunit
414	P17931	Galectin-3
415	P17987	T-complex protein 1 subunit alpha
416	P18124	60S ribosomal protein L7
417	P19338	Nucleolin
418	P19367	Hexokinase-1
419	P19623	Spermidine synthase
420	P20073	Annexin A7
421	P21796	Voltage-dependent anion-selective channel protein
422	P22087	rRNA 2'-O-methyltransferase fibrillarin
423	P23246	Splicing factor, proline- and glutamine-rich
424	P23368	NAD-dependent malic enzyme, mitochondrial
425	P23381	Tryptophan--tRNA ligase, cytoplasmic
426	P23526	Adenosylhomocysteinase
427	P24752	Acetyl-CoA acetyltransferase, mitochondrial
428	P25398	40S ribosomal protein S12
429	P25799	NFκB
430	P26038	Moesin
431	P26639	Threonine--tRNA ligase, cytoplasmic
432	P26641	Elongation factor 1-gamma
433	P27824	Calnexin
434	P28838	Cytosol aminopeptidase
435	P30044	Peroxiredoxin 5, mitochondrial
436	P30048	Thioredoxin-dependent peroxide reductase, mitochondrial
437	P30050	60S ribosomal protein L12
438	P30084	Enoyl-CoA hydratase, mitochondrial
439	P30101	Protein disulfide-isomerase A
440	P31150	Rab GDP dissociation inhibitor
441	P31939	Bifunctional purine biosynthesis protein PURH
442	P31947	14-3-3 protein sigma
443	P35270	Sepiapterin reductase
444	P35998	26S protease regulatory subunit 7

445	P36952	Serpin B5
446	P37802	Transgelin-2
447	P38117	Electron transfer flavoprotein subunit beta
448	P38646	Stress-70 protein, mitochondrial
449	P39748	Flap endonuclease 1
450	P40227	T-complex protein 1 subunit zeta
451	P41091	Eukaryotic translation initiation factor 2 subunit 3
452	P41250	Glycine--tRNA ligase
453	P42574	Caspase 3
454	P43686	26S protease regulatory subunit 6B
455	P46783	40S ribosomal protein S10
456	P47756	F-actin-capping protein subunit beta
457	P48735	Isocitrate dehydrogenase [NADP], mitochondrial
458	P48741	Putative heat shock 70 kDa protein 7
459	P49189	4-trimethylaminobutyraldehyde dehydrogenase
460	P49411	Elongation factor Tu, mitochondrial
461	P49588	Alanine--tRNA ligase, cytoplasmic
462	P49748	Very long-chain specific acyl-CoA dehydrogenase, mitochondrial
463	P50990	T-complex protein 1 subunit theta
464	P50991	T-complex protein 1 subunit delta
465	P51991	Heterogeneous nuclear ribonucleoprotein A3
466	P52789	Hexokinase-2
467	P53004	Biliverdin reductase A
468	P53396	ATP-citrate synthase
469	P53621	Coatomer subunit alpha
470	P54136	Arginine--tRNA ligase, cytoplasmic
471	P54886	Delta-1-pyrroline-5-carboxylate synthase
472	P55010	Eukaryotic translation initiation factor 5
473	P55060	Exportin-2
474	P56537	Eukaryotic translation initiation factor 6
475	P60228	Eukaryotic translation initiation factor 3 subunit E
476	P60866	40S ribosomal protein S20
477	P61204	ADP-ribosylation factor 3
478	P61254	60S ribosomal protein L26
479	P61978	Heterogeneous nuclear ribonucleoprotein K
480	P61981	14-3-3 protein gamma
481	P62136	Serine/threonine-protein phosphatase PP1
482	P62195	26S protease regulatory subunit 8
483	P62258	14-3-3 protein epsilon
484	P62308	Small nuclear ribonucleoprotein G
485	P62701	40S ribosomal protein S4, X isoform
486	P62851	40S ribosomal protein S25
487	P62854	40S ribosomal protein S26
488	P62906	60S ribosomal protein L10a
489	P62913	60S ribosomal protein L11

490	P63162	Small nuclear ribonucleoprotein-associated protein N
491	P63261	Actin, cytoplasmic 2
492	P68366	Tubulin alpha-4A
493	P68371	Tubulin beta-4B
494	P78527	DNA-dependent protein kinase catalytic subunit
495	P84077	ADP-ribosylation factor 1
496	P84098	60S ribosomal protein L19
497	Q00839	Heterogeneous nuclear ribonucleoprotein U
498	Q01518	Adenylyl cyclase-associated protein 1
499	Q01813	ATP-dependent 6-phosphofructokinase
500	Q05639	Elongation factor 1-alpha 2
501	Q05BS0	Eukaryotic translation initiation factor 3 subunit A
502	Q05CK9	SYNCRIP protein
503	Q05CV6	CPS1 protein
504	Q05D48	TNPO2 protein
505	Q06210	Glutamine--fructose-6-phosphate aminotransferase
506	Q07020	Ribosomal protein S4
507	Q07021	Complement component 1 Q subcomponent-binding protein, mitochondrial
508	Q08170	Serine/arginine-rich splicing factor 4
509	Q08211	ATP-dependent RNA helicase A
510	Q08AJ6	ANP32A protein
511	Q08ES8	Cell growth-inhibiting protein 34
512	Q0D2M2	HIST1H2BC protein
513	Q0EFC9	TC4 protein
514	Q0IIN5	VCP protein
515	Q0IJ56	ST13 protein
516	Q0QER2	Isocitrate dehydrogenase 1
517	Q0QEW2	Ribosomal protein L18
518	Q0VAS5	Histone H4
519	Q0VGA5	SARS protein
520	Q0VGD6	HNRPR protein
521	Q0VGL3	RPL13A protein
522	Q13041	P67
523	Q13247	Serine/arginine-rich splicing factor 6
524	Q13509	Tubulin beta-3
525	Q13748	Tubulin alpha-3C/D
526	Q13867	Bleomycin hydrolase
527	Q13885	Tubulin beta-2A
528	Q14103	Heterogeneous nuclear ribonucleoprotein D0
529	Q14204	Cytoplasmic dynein 1 heavy chain 1
530	Q14315	Filamin C
531	Q14666	Radiated keratinocyte mRNA 266
532	Q14914	Prostaglandin reductase 1
533	Q14917	SPTAN1 protein
534	Q15008	26S proteasome non-ATPase regulatory subunit 6

535	Q15029	116 kDa U5 small nuclear ribonucleoprotein component
536	Q15056	Eukaryotic translation initiation factor 4H
537	Q15374	GARS protein
538	Q15393	Splicing factor 3B subunit 3
539	Q15717	ELAV-like protein 1
540	Q16236	Nuclear factor erythroid 2-related factor 2
541	Q16401	26S proteasome non-ATPase regulatory subunit 5
542	Q16444	Phosphoglycerate kinase
543	Q16555	Dihydropyrimidinase-related protein 2
544	Q16658	Fascin
545	Q1AHP8	Hepatopoietin PCn127
546	Q1KLZ0	HCG15971
547	Q1XBU6	Aging-associated protein 14b
548	Q2F838	Eukaryotic translation elongation factor 1 gamma
549	Q2F839	Heat shock 70 kDa protein 9B
550	Q2NKY5	TUBB6 protein
551	Q2TNB3	Cell migration-inducing protein 22
552	Q2TSD0	Glyceraldehyde-3-phosphate dehydrogenase
553	Q2TU84	Aspartate aminotransferase
554	Q2TUW9	Lactoferrin
555	Q2VIN3	RBM1
556	Q2VIR3	Putative eukaryotic translation initiation factor 2
557	Q2VPA0	Lon protease
558	Q2VPJ6	HSP90AA1 protein
559	Q2XPP3	Type II 3a-hydroxysteroid dehydrogenase
560	Q2YD88	FAM129B protein
561	Q32Q12	Nucleoside diphosphate kinase
562	Q3B792	NQO1 protein
563	Q3B7A3	SEPT7 protein
564	Q3KNR6	Hsc70-interacting protein
565	Q3KQZ8	EPRS protein
566	Q3MIH3	Ubiquitin A-52
567	Q3ZCR3	TUBB3 protein
568	Q49A90	RPS27A protein
569	Q49AJ9	RPL3 protein
570	Q49AK0	LTA4H protein
571	Q49AN9	SNRPG protein
572	Q4JM47	AGR2
573	Q4LE36	ACLY variant protein
574	Q4LE58	EIF4G1
575	Q4LE60	TNPO2 protein
576	Q4VB24	Histone cluster 1
577	Q52NV4	Histidyl-tRNA synthetase
578	Q53EM5	Transketolase
579	Q53F35	Acidic (Leucine-rich) nuclear phosphoprotein 32 family, member B variant

580	Q53F45	Splicing factor, arginine/serine-rich 4
581	Q53FB0	Chloride intracellular channel protein
582	Q53FG3	Interleukin enhancer binding factor 2
583	Q53FN7	BZW1 protein variant
584	Q53FW2	Phosphoribosyl pyrophosphate synthetase 1
585	Q53G58	Coronin
586	Q53G71	Calreticulin
587	Q53G74	Ribosomal protein L4
588	Q53G81	NAD(P)H menadione oxidoreductase 1
589	Q53G83	Ribosomal protein S3
590	Q53G92	Tubulin, beta 4
591	Q53GA7	Tubulin alpha 6
592	Q53GL5	Isocitrate dehydrogenase 2
593	Q53GN4	WD repeat domain 1
594	Q53GN6	Proteasome 26S non-ATPase subunit
595	Q53GX7	Threonyl-tRNA synthetase v
596	Q53GZ6	Heat shock 70kDa protein 8
597	Q53H17	WD repeat-containing protein 1
598	Q53H34	Ribosomal protein L13
599	Q53HA4	Seryl-tRNA synthetase
600	Q53HE7	Small nuclear ribonucleoprotein polypeptide N
601	Q53HR2	Acyl-Coenzyme A dehydrogenase
602	Q53HS0	Glutaminyl-tRNA synthetase
603	Q53HU0	Chaperonin containing TCP1, subunit 8 (Theta) variant
604	Q53HV2	Chaperonin containing TCP1, subunit 7
605	Q53SS8	Epididymis secretory protein Li 85
606	Q53XL8	Proteasome 26S subunit
607	Q53Y51	D-dopachrome tautomerase
608	Q569J8	HNRPC protein
609	Q58F26	DHX9 protein
610	Q58FF2	Heat shock protein 94c
611	Q58FF3	Putative endoplasmin-like protein
612	Q58FF9	Heat shock protein 90Af
613	Q59EF6	Calpain 2
614	Q59EJ0	Aldehyde dehydrogenase 1A1
615	Q59EJ3	Heat shock 70kDa protein 1A
616	Q59EJ5	Glutathione S-transferase M3
617	Q59EM6	Internexin neuronal intermediate filament protein
618	Q59ET0	Glucan , branching enzyme 1 variant
619	Q59ET3	Chaperonin containing TCP1, subunit 6A
620	Q59EY4	CDC10 protein variant
621	Q59F66	DEAD box polypeptide 17 isoform p82
622	Q59F68	CD68 antigen
623	Q59F85	Glucose phosphate isomerase
624	Q59FF0	EBNA-2 co-activator

625	Q59FH0	H2A histone family, member Y
626	Q59FI9	Ribosomal protein L12
627	Q59FV6	ARP3 actin-related protein 3
628	Q59G24	Activated RNA polymerase II transcription cofactor 4 variant
629	Q59G78	Lactate dehydrogenase B
630	Q59GB4	Dihydropyrimidinase-like 2
631	Q59GE4	Ribosomal protein S10
632	Q59GF8	Heat shock 70kDa protein 4
633	Q59GK9	Ribosomal protein L21
634	Q59GL1	Synaptotagmin binding, cytoplasmic RNA interacting protein
635	Q59GP7	Interleukin enhancer binding factor 3
636	Q59GR8	TPM1 protein
637	Q59GX6	DEAD/H (Asp-Glu-Ala-Asp/His) box polypeptide 3
638	Q59GY3	Arginine/serine-rich splicing factor 6 variant
639	Q59HA3	IQ motif containing GTPase activating protein 2
640	Q59HH3	Phosphoribosylglycinamide formyltransferase
641	Q5CAQ4	TNF receptor-associated protein 1
642	Q5H928	3-hydroxyacyl-CoA dehydrogenase type-2
643	Q5HYB6	Epididymis luminal protein 189
644	Q5JNZ5	Putative 40S ribosomal protein
645	Q5JR95	40S ribosomal protein S8
646	Q5JRR6	Ubiquitin-like modifier-activating enzyme 1
647	Q5M7Z9	TARS protein
648	Q5QTS3	FWP004
649	Q5R211	Carbamoyl-phosphate synthase
650	Q5RLN2	Desmin
651	Q5SU16	Beta 5-tubulin
652	Q5SYQ9	Retinal dehydrogenase 1
653	Q5T3N1	Annexin
654	Q5T5C7	Serine--tRNA ligase, cytoplasmic
655	Q5T7N0	60S ribosomal protein L5
656	Q5T8F0	Cathepsin L1
657	Q5T8U2	60S ribosomal protein L7a
658	Q5TA02	Glutathione S-transferase omega-1
659	Q5TB19	Acidic (Leucine-rich) nuclear phosphoprotein 32 family, member E
660	Q5TB52	3'-phosphoadenosine 5'-phosphosulfate synthase 2
661	Q5TCD1	Isoleucine--tRNA ligase, cytoplasmic
662	Q5TCI8	Prelamin-A/C
663	Q5TEC6	Histone H3
664	Q5VTE0	Putative elongation factor 1-alpha-like 3
665	Q5VU62	TPM3 protein
666	Q60FE5	Filamin A
667	Q65ZQ3	FBRNP
668	Q66K91	Small nuclear ribonucleoprotein-associated protein
669	Q6DN03	Putative histone H2B

670	Q6FHK8	PGAM1 protein
671	Q6FHP5	PHB protein
672	Q6FHU2	Epididymis secretory protein Li 35
673	Q6FHV6	ENO2 protein
674	Q6FI35	Proliferating cell nuclear antigen
675	Q6I9Y8	IPO4 protein
676	Q6IAX2	RPL21 protein
677	Q6IBA2	PC4 protein
678	Q6IBG5	MYL6 protein
679	Q6IBH6	RPL26 protein
680	Q6IBM8	U5-116KD protein
681	Q6IBN0	PSMD3 protein
682	Q6IBN1	HNRPK protein
683	Q6IBR2	FARSLA protein
684	Q6IBS5	DLST protein
685	Q6IBT3	CCT7 protein
686	Q6IBU0	EIF5 protein
687	Q6IC76	G22P1 protein
688	Q6IN67	HYOU1 protein
689	Q6IPF2	Heterogeneous nuclear ribonucleoprotein A1
690	Q6IPH7	RPL14 protein
691	Q6LBS1	SmB /B' autoimmune antigene
692	Q6LC01	MRNA encoding beta-tubulin
693	Q6LET3	HPRT1 protein
694	Q6NSF2	RPLP0 protein
695	Q6NVY0	Calcyclin binding protein
696	Q6NWZ1	CKAP4 protein
697	Q6P1L4	PYGL protein
698	Q6P1N4	IQGAP1 protein
699	Q6P2H7	DYNC1H1 protein
700	Q6P2Q9	Pre-mRNA-processing-splicing factor 8
701	Q6P4B4	PFAS protein
702	Q6P4C9	EEF1A1 protein
703	Q6PCE3	Glucose 1,6-bisphosphate synthase
704	Q6PEY2	Tubulin alpha-3E
705	Q6PIN5	PA2G4 protein
706	Q6PIX2	SFPQ protein
707	Q6PJ43	ACTG1 protein
708	Q6PJJ3	FASN protein
709	Q6PJT4	MSN protein
710	Q6PJY1	FUBP1 protein
711	Q6PK50	HSP90AB1 protein
712	Q6PKA6	ALDH3A1 protein
713	Q6PUJ7	Epididymis luminal protein 215
714	Q6QE17	IMP dehydrogenase 2

715	Q6QMJ5	Tubulin alpha 1
716	Q6S8J3	POTE ankyrin domain family member E
717	Q6ZNL4	FLJ00279 protein
718	Q71RH4	FP1047
719	Q75MH1	HCG1745083
720	Q76LA1	CSTB protein
721	Q7KYM9	ORF protein
722	Q7KZF4	Staphylococcal nuclease domain-containing protein 1
723	Q7KZX8	G1 to S phase transition 1
724	Q7L4K8	IARS protein
725	Q7L4M3	KRT8 protein
726	Q7Z3X3	N-acetylglucosamine-6-sulfatase
727	Q7Z474	Proteasome subunit beta
728	Q7Z4Y3	Arsenite-resistance protein ARS2
729	Q7Z726	Importin
730	Q7Z759	CCT8 protein
731	Q86UY0	TXNDC5 protein
732	Q86VG2	Splicing factor proline/glutamine-rich
733	Q86WD0	Eukaryotic translation initiation factor 4A isoform 2-like protein
734	Q86XU5	MYH9 protein
735	Q8IUB0	CTCL tumor antigen HD-CL-08
736	Q8IZ29	Tubulin, beta 2C
737	Q8J015	60S ribosomal protein L13a
738	Q8N163	Cell cycle/apoptosis regulator protein 2
739	Q8N1C8	HSPA9 protein
740	Q8N532	TUBA1C protein
741	Q8N5M8	PHGDH protein
742	Q8NBS9	Thioredoxin domain-containing protein 5
743	Q8NBX0	Saccharopine dehydrogenase-like oxidoreductase
744	Q8NCW5	NAD(P)H-hydrate epimerase
745	Q8TCG4	TPMsk1
746	Q8TD11	Decay-accelerating factor 4ab
747	Q8TD12	Decay-accelerating factor 3
748	Q8TD13	Decay-accelerating factor 1 ab
749	Q8TD47	40S ribosomal protein S4, Y isoform
750	Q8TES4	FLJ00119 protein
751	Q8VDP4	Cell cycle and apoptosis regulator protein 1
752	Q8WUI6	COPG protein
753	Q8WVX7	Ribosomal protein S19
754	Q8WYN9	Ribosomal protein S27
755	Q92688	Acidic leucine-rich nuclear phosphoprotein 32 family member B
756	Q96AY3	Peptidyl-prolyl cis-trans isomerase
757	Q96B07	EIF4A2 protein
758	Q96BA7	HNRPU protein
759	Q96BG6	ACTN4 protein

760	Q96BS4	FBL protein
761	Q96C19	EF-hand domain-containing protein D2
762	Q96C32	Polyubiquitin-C
763	Q96C61	FLNA protein
764	Q96DG6	Carboxymethylenebutenolidase
765	Q96E39	RNA binding motif protein, X-linked-like-1
766	Q96G03	Phosphoglucomutase-2
767	Q96H31	UBC protein
768	Q96HE7	ERO1-like protein
769	Q96HX0	TUBB2C protein
770	Q96IR1	RPS4X protein
771	Q96IS6	HSPA8 protein
772	Q96KP4	Cytosolic non-specific dipeptidase
773	Q96QL0	Ribosomal protein L3
774	Q96RE1	Translation elongation factor 1 alpha 1-like 14
775	Q99832	T-complex protein 1 subunit eta
776	Q9BQ02	NCL protein
777	Q9BR63	FARSB protein
778	Q9BS14	GANAB protein
779	Q9BS26	Endoplasmic reticulum resident protein 44
780	Q9BTI9	NPM1 protein
781	Q9BUF5	Tubulin beta-6
782	Q9BUV4	RPL5 protein
783	Q9BUZ3	QARS protein
784	Q9BV61	TRAP1 protein
785	Q9BVA1	Tubulin beta-2B
786	Q9BXP5	Serrate RNA effector molecule homolog
787	Q9BYN0	Sulfiredoxin-1
788	Q9BZ93	Prosomal P27K protein
789	Q9H2U2	Inorganic pyrophosphatase 2, mitochondrial
790	Q9H369	PRO1633
791	Q9H3A5	PRO2195
792	Q9H7K8	FLJ00064 protein
793	Q9HAP0	Valosin-containing protein
794	Q9HC38	Glyoxalase domain-containing protein 1
795	Q9NR30	Nucleolar RNA helicase 2
796	Q9NR45	Sialic acid synthase
797	Q9NYD7	High mobility group 1 protein
798	Q9NYF8	Bcl-2-associated transcription factor 1
799	Q9NZ23	Drug-sensitive protein 1
800	Q9NZE6	BM-010
801	Q9NZM1	Myoferlin
802	Q9P0M6	Core histone macro-H2A.2
803	Q9P1G4	PRO1837
804	Q9P1N9	PRO0785

805	Q9P2J5	Leucine--tRNA ligase, cytoplasmic
806	Q9UIR2	ATP sulfurylase/APS kinase isoform SK2
807	Q9UKK9	ADP-sugar pyrophosphatase
808	Q9UMY3	3-phosphoglycerate dehydrogenase
809	Q9UN47	Frameshifted pyruvate kinase M2
810	Q9UNM1	Chaperonin 10-related protein
811	Q9UNM7	26S proteasome subunit p40.5
812	Q9UNN8	Endothelial protein C
813	Q9UQC1	Heat shock protein 72
814	Q9UQM3	Alpha-tubulin
815	Q9Y230	RuvB-like 2
816	Q9Y265	RuvB-like 1
817	Q9Y2B0	Protein canopy homolog 2
818	Q9Y376	Calcium-binding protein 39
819	Q9Y3E8	CGI-150 protein
820	Q9Y678	Coatomer subunit gamma-1
821	Q9Y6E3	HSPC027
822	R4GMR5	26S proteasome non-ATPase regulatory subunit 8
823	R4GN08	Actin-related protein 2/3 complex subunit 4
824	R4SBI6	EPHX1
825	S4R3N1	Protein HSPE1-MOB4
826	U3KQ84	Dolichyl-diphosphooligosaccharide--protein glycosyltransferase
827	V9GYZ6	Bifunctional glutamate/proline--tRNA ligase
828	V9GZ37	Heat shock 70 kDa protein 1A
829	V9HVX6	Epididymis luminal protein 9
830	V9HVZ0	Epididymis secretory protein Li 91
831	V9HVZ7	Epididymis luminal protein 176
832	V9HW22	Epididymis luminal protein 33
833	V9HW24	Epididymis secretory protein Li 73
834	V9HW25	Epididymis secretory protein Li 273
835	V9HW26	ATP synthase subunit alpha
836	V9HW31	ATP synthase subunit beta
837	V9HW35	Epididymis secretory protein Li 55
838	V9HW37	Epididymis secretory protein Li 69
839	V9HW38	Epididymis secretory protein Li 106
840	V9HW41	Epididymis secretory protein Li 71
841	V9HW42	Epididymis secretory protein Li 105
842	V9HW44	Epididymis secretory protein Li 303
843	V9HW55	Epididymis secretory protein Li 275
844	V9HW69	Epididymis secretory protein Li 66
845	V9HW77	Epididymis luminal protein 211
846	V9HW79	D-3-phosphoglycerate dehydrogenase
847	V9HW80	Epididymis luminal protein 220
848	V9HW90	Epididymis luminal protein 75
849	V9HWB8	Pyruvate kinase

850	V9HWC9	Superoxide dismutase [Cu-Zn]
851	V9HWD6	Epididymis secretory protein Li 1
852	V9HWE1	Epididymis luminal protein 113
853	V9HWE9	Epididymis secretory protein Li 22
854	V9HWG9	Epididymis secretory protein Li 21
855	V9HWH1	Epididymis luminal protein 57
856	V9HWH2	Creatine kinase brain isoform 1
857	V9HWH9	Protein S100
858	V9HWI4	Epididymis luminal protein 110
859	V9HWK1	Triosephosphate isomerase
860	V9HWK2	Epididymis luminal protein 114
861	V9HWK4	Epididymis luminal protein 162
862	X6RA14	S-formylglutathione hydrolase
